# Severe infections caused by difficult-to-treat Gram-negative bacteria

**DOI:** 10.1097/MCC.0000000000001074

**Published:** 2023-07-31

**Authors:** Silvia Dettori, Federica Portunato, Antonio Vena, Daniele Roberto Giacobbe, Matteo Bassetti

**Affiliations:** a Infectious Diseases Unit, San Martino Policlinico Hospital - IRCCS for Oncology and Neuroscience; b Department of Health Sciences (DISSAL), University of Genoa, Genoa, Italy

**Keywords:** *Acinetobacter baumannii*, difficult-to-treat resistance, DTR Enterobacterales, Gram-negative bacteria, *Pseudomonas aeruginosa*

## Abstract

**Purpose of review:**

Antimicrobial resistance (AMR) in Gram-negative bacteria (GNB) poses a significant global health concern, contributing to increased infections, mortality rates, and healthcare costs. This review discusses the main clinical manifestations, therapeutic options, and recent findings in managing antibiotic-resistant GNB, with a focus on difficult-to-treat infections.

**Recent findings:**

Difficult-to-treat resistance (DTR) is a novel classification that identifies GNB exhibiting intermediate or resistant phenotypes to first-line agents in the carbapenem, beta-lactam, and fluoroquinolone categories. The main pathogens implicated in severe infections include DTR Enterobacterales, DTR *Pseudomonas aeruginosa*, and DTR *Acinetobacter baumannii.* Although the clinical implications of DTR strains are still under investigation, certain studies have linked them to prolonged hospital stays and poor patient outcomes.

**Summary:**

Severe infections caused by DTR-GNB pose a formidable challenge for healthcare providers and represent a growing global health issue. The proper administration and optimization of novel antibiotics at our disposal are of paramount importance for combating bacterial resistance and improving patient prognosis.

## INTRODUCTION

The emergence of antimicrobial resistance (AMR) in Gram-negative bacteria (GNB) is an evolving public health concern, that is causing a surge in the incidence of infections, mortality rates, and associated healthcare costs. In the United States, the financial burden of AMR was estimated to be approximately 2.4 billion dollars in 2017 [1,2]. Globally, AMR was responsible for nearly 4.95 million deaths in 2019, with GNB accounting for 10 out of the 18 strains highlighted in the Centers for Disease Control (CDC) and Prevention's threats report [3,4].

Traditionally, AMR has been classified from the United States and European CDC and Prevention (ECDC) into three types: multidrug resistance (MDR), extensive drug resistance (XDR), and pan-drug resistance (PDR) [5]. However, the utility of this classification in clinical practice has been questioned mainly because it does not correlate with clinical outcomes [6,7].

In 2018, Kadri *et al.* proposed a new definition for the resistance profile of GNB, known as difficult-to-treat resistance (DTR) [8]. DTR refers to an intermediate or resistant phenotype to all first-line agents in the carbapenem, beta-lactam, and fluoroquinolone categories. The term focuses on nonsusceptibility to all first-line, high-efficacy, and low-toxicity agents, considering the impact of resistance on treatment decisions and clinical outcomes [8]. Although the new definition is currently under debate, its association with clinical outcomes is being evaluated [8–10]. Hopefully, with the introduction and proper use of new antibiotics, DTR can be effectively managed in the future.

This review provides an overview of the main clinical manifestations associated with DTR-GNB infections in the hospital setting, as well as the latest available antibiotics for these challenging infections. 

**Box 1:**
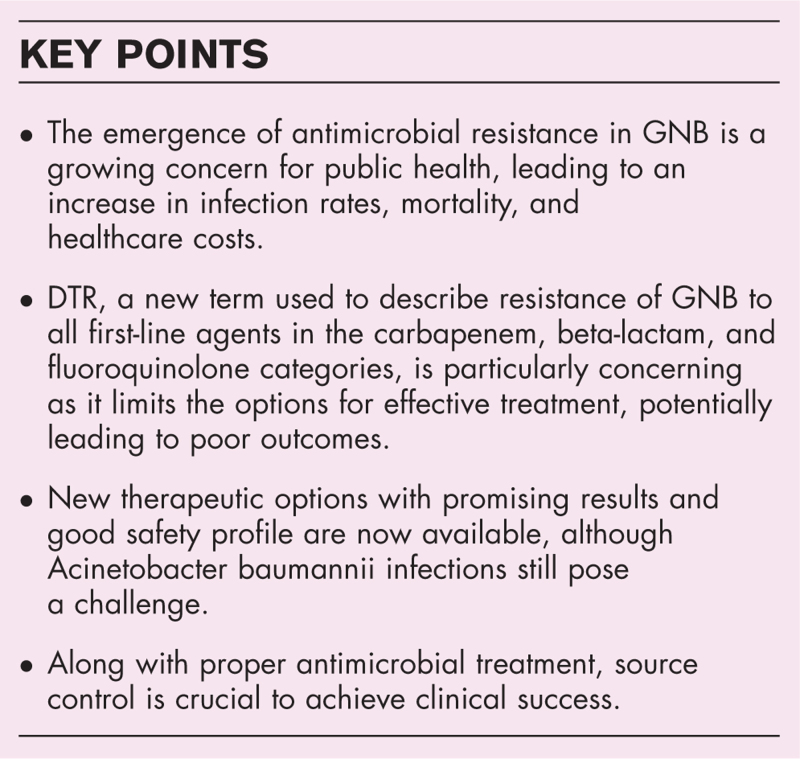
no caption available

## CLINICAL MANIFESTATIONS

Gram-negative bacteria (GNB) are associated with various severe nosocomial infections, including hospital-acquired pneumonia (HAP)/ventilator-associated pneumonia (VAP), bloodstream infections (BSI), and urinary tract infections (UTI). In the EPIC-III survey and in the most recent EUROBACT-2 on infections in ICUs, GNB infections accounted for over 60% of cases, with more than 24% of strains classified as DTR [9,10]. In this section, we will discuss the main clinical manifestations caused by DTR-GNB.

### Nosocomial pneumonia

Nosocomial pneumonia, including HAP and VAP, represents one of the most frequent and serious healthcare-associated infections [9,10]. The prevalence of HAP is reported to be from 5 to 20 cases per 1000 hospital admissions, with about two-third of cases represented by nonventilated HAP. VAP prevalence ranges from 2 to 21 per 1000 invasive mechanical ventilation days, affecting up to 25% of ventilated patients, with a peak of 50% in patients with head trauma [11–13].

In the ICU setting, VAP represent 60% of total infections, with the main pathogens identified being primarily GNB (such as *Klebsiella pneumoniae*, *Pseudomonas aeruginosa*, and *Acinetobacter baumannii*), with a MDR profile in about half of cases [9,11,14]. Nosocomial pneumonia is associated with poor outcomes, with VAP crude mortality varying across studies from 15 to 50%, and directly related death up to 19%. Moreover, ventilated HAP represents an important entity with a mortality rate of 28%, compared with 15% for nonventilated HAP [14,15].

In a recent study, HAP required mechanical ventilation in about 38% of cases with an increased risk of mortality of 82 compared with 38% of VAP [13,16]. The causative agent also has a prognostic impact, with ICU mortality significantly higher in patients with resistant strains than in those without (47.5 vs. 28.7%). Additionally, ICU length of stay and duration of mechanical ventilation were increased in patients infected with DTR pathogens [14,17].

### Bloodstream infections

Overall, BSIs account for about 40% of community-acquired and hospital-acquired infections, causing sepsis and septic shock. In critically ill patients, 75% of BSIs are ICU-acquired, whereas 25% are documented at ICU admission. Different factors contribute to an increased risk of BSI during hospital stay, including invasive manoeuvres, such as central venous catheters (CVC) [18]. As reported in the EUROBACT-2 study, among patients admitted to the ICU, the most common sources of BSI were the CVC and the lung (26% of cases each), whereas 16% had an unknown origin [10].

GNB are responsible for most BSIs, both in the community and hospital settings, with *Escherichia coli, K. pneumoniae*, and *P. aeruginosa* being the most frequent isolates. *P. aeruginosa* and *A. baumannii* are more often isolated in hospital-acquired infections [19].

Using data from a well distributed sample of US hospitals, Kadri *et al.* determined that the 5-year prevalence of DTR among GNB BSI was 1%, with more than 70% of those episodes occurring in patients admitted to the ICU. In this study, DTR was identified in 18% of *A. baumannii*, 2.3% of *P. aeruginosa*, and 1.7% of *K. pneumoniae* BSI episodes [8]. Recent data from the EUROBACT-2 study reported that GNB caused 59% of BSIs, with *Klebsiella* spp. and *Acinetobacter* spp. being the most frequently reported, and DTR-GNB accounting for 24% of isolates [10]. Similar data were reported in an Italian study: among patients hospitalized with nonfermentative GNB BSI, DTR prevalence was 14 and 64% in *P. aeruginosa* and *A. baumannii* isolates, respectively. In this cohort, BSIs were catheter-related in 29% of cases and had an unknown origin in about half of the cases [20]. In patients with BSIs caused by DTR-GNB, mortality increased up to 50%, and the risk of death was increased by 40% compared with patients without resistant strains [2,8,10,21].

### Intra-abdominal infections

Intra-abdominal infections (IAIs) represent the third most common infection in critically ill patients, accounting for 17% of ICU infections, following pneumonia and catheter-related bloodstream infections (CR-BSI) [9,10]. Postoperative peritonitis is the most frequent type of IAI in ICU patients (65% of cases) [22]. A large survey reported that 32% of IAIs in critically ill patients were community-acquired, 25% were early-onset hospital-acquired (<7 days from hospital admission), whereas 43% were late-onset hospital-acquired (≥7 days from admission), the latter being associated with worse severity and more often related to septic shock [23].

GNB are the main pathogen responsible for IAIs, representing more than half of microbiological isolates (around 15% in upper gastrointestinal tract perforation, reaching 80% in intestinal/colon-related peritonitis), and Enterobacterales being the predominant group. Nonfermentative GNB-like *P. aeruginosa* and *A. baumannii* are often responsible for tertiary peritonitis, occurring in patients requiring multiple surgical revisions [22–24]. The rate of resistant strains in IAIs ranges from 26 to 65% in different reports [23,25–27]. In the AbSeS study, DTR-GNB represented 4.3% of the total [23]. Length of hospital stay prior to ICU admission, surgical re-exploration, and previous treatment with carbapenems and fluoroquinolones were found to be significantly correlated with an increased risk for resistant GNB [25].

Overall mortality for IAIs in critically ill patients ranges from 29 to 42%, being higher for late-onset hospital-acquired IAIs, especially when septic shock is present [23,28,29]. Other independent factors that have an impact on mortality are older age, failure of source control, diffuse peritonitis, and infection with resistant strains, with the latter being the most important, associated higher in-hospital mortality (56%) and longer hospital stay compared to patients without [25,30].

### Urinary tract infections

UTIs are a predominant cause of infection both in the community and in the hospital setting, resulting in sepsis or septic shock in up to 25% of cases [31]. In hospitalized patients, UTIs are frequently related to the presence of urinary catheters (CAUTI) with an incidence of 8.9/1000 catheter days; critically ill patients are considerably exposed to this risk because of the prolonged duration of catheterization [32]. In the ICU, UTIs represent about 11% of infections [9]. Moreover, in this setting, the differential diagnosis between asymptomatic bacteriuria and infection could be extremely challenging due to the absence of symptoms and the presence of other possible sources of infection. It has been noted that bacteriuria increases by 3–7%/day in patients with indwelling urinary catheters [32].

The most common causative pathogens are usually Enterobacterales, especially *E. coli* and *K. pneumoniae*, but in patients with CAUTI, *P. aeruginosa* and *Enterococcus* spp. are also frequently isolated. Due to the rapid spread of resistance phenotypes, DTR-GNB are responsible for 22% of UTI-associated BSI [8,33]. Patients with UTI had an increased length of hospital stay, with ICU and in-hospital mortality being 22 and 29%, respectively [9,33].

## ANTIBIOTIC THERAPY FOR DIFFICULT-TO-TREAT RESISTANCE GRAM-NEGATIVE BACTERIA INFECTIONS

Early and appropriate treatment is considered the cornerstone for improving clinical outcomes in patients with severe infections [34]. This concept is particularly important in patients with DTR-GNB severe infections, where delayed appropriate antimicrobial treatment has been associated with higher mortality rates [35–37]. First-line agents are inadequate for this type of infections, and more complex therapeutic options are needed. Until recently, the only options were polymyxins in combination with tetracyclines, Fosfomycin, or aminoglycosides. However, these regimens were associated with significant toxicity and low tissue concentration, with very low response rates [38,39].

New antibiotics are now available, mostly belonging to the beta-lactams/beta-lactams inhibitor (BL/BLI) class, and demonstrated high clinical success rates with good safety profiles compared with older options [40^▪^]. Empirical treatment with new agents should be based on the risk factors for DTR-GNB infections. Recent hospitalization, recent antibiotic therapy, indwelling devices, and prior colonization may represent some of the most important predisposing conditions to be evaluated [41].

Despite the current variety of treatment options, selecting the appropriate antibiotic for empirical therapy remains challenging, and considerations for the supposed resistance mechanism to be covered is crucial.

### Difficult-to-resistance Enterobacterales

Although several mechanisms can cause a difficult-to-treat phenotype (e.g. porin loss, efflux pumps, or beta-lactamase production), in most Enterobacterales strains, the main mechanism of antibiotic resistance is the production of Class A (e.g. KPC) and Class D carbapenemases (e.g. OXA-type) [42]. Additionally, class B (including metallo-beta-lactamase – MBL) is also a significant mechanisms of resistance. Given the increasing spread of these enzymes, it is crucial to be aware of local epidemiology and patient colonization to guide an adequate empiric therapy.

Current guidelines recommend ceftazidime/avibactam and meropenem/vaborbactam as first-line agents for the treatment of severe infections because of DTR Enterobacterales producing class A carbapenemase, whereas imipenem/relebactam and cefiderocol as alternatives. Ceftazidime/avibactam is undoubtedly the first option for infections caused by OXA-48-producing Enterobacterales, with cefiderocol as second choice [43^▪^–45^▪^]. Real-life experience shows better clinical outcomes, with up to 64% success rates and 18% mortality in patients receiving ceftazidime/avibactam for DTR Enterobacterales infections compared with older options [46–50].

Meropenem/vaborbactam is another promising option, with the TANGO-II phase 3 study demonstrating higher clinical cure rate and decreased mortality compared with older treatments [51,52]. Moreover, it is an interesting option for DTR-GNB pneumonia because of its higher epithelial lining fluid concentrations, and it can be considered in some cases of ceftazidime/avibactam resistance [53,54].

Imipenem/relebactam has shown promising activity against class A serine-carbapenemase *in vitro*[55–57] and, to the best of our knowledge, few studies are currently available for its use in DTR Enterobacterales infections [58,59].

Cefiderocol also demonstrated strong activity against DTR Enterobacterales and is one of the best options when MBL enzymes are expressed, as highlighted in a sub-analysis of the CREDIBLE-CR and APEKS-NP registration trials [60^▪^]. However, resistance may be present in New Delhi MBL (NDM)-producing strains [61–63]. Currently, the first option for MBL-producing Enterobacterales is ceftazidime/avibactam in combination with aztreonam, which has shown high efficacy and significantly lower mortality rates compared with polymyxin or tetracycline-based regimens in real-life experience [64,65]. Imipenem-relebactam plus aztreonam also showed promising in-vitro activity for MBL-producing strains but more clinical data are needed [66,67^▪▪^].

Finally, eravacycline, a novel synthetic fluorocycline, could be considered for DTR Enterobacterales infections, but only for IAI, where it has shown noninferiority compared with ertapenem and meropenem in the IGNITE-1 and IGNITE-4 trials [68,69].

### Difficult-to-resistance *Pseudomonas aeruginosa*

DTR *P. aeruginosa* represents a significant challenge for clinicians, as it can exhibit various resistance mechanisms, including upregulation of efflux pumps, loss or reduction of outer membrane porins (OprD), hyperproduction of AmpC enzymes, and mutations of penicillin-binding proteins [70,71]. Although carbapenemase production was previously less frequent, there has been a recent increase in prevalence, with a worrisome expression of MBL [72,73].

Ceftolozane/tazobactam, ceftazidime/avibactam, and imipenem/relebactam are the preferred regimens for empiric treatment of DTR *P. aeruginosa*, although antibiotic susceptibility testing is necessary, considering the growing number of strains resistant to new antibiotics [43^▪^–45^▪^].

Ceftolozane/tazobactam has shown very high clinical cure rates in real-world experience, despite a small number of DTR *P. aeruginosa* infections being treated with this drug in the ASPECT-NP trial compared with meropenem [74–78]. Furthermore, ceftolozane/tazobactam has demonstrated lower side effects than regimens containing polymyxins or aminoglycosides [79,80]. High success rates, up to 83%, have been achieved in real-life experience, evaluating different types of infections because of *P. aeruginosa*, including critically ill patients, with over 50% of *P. aeruginosa* strains being resistant [81,82]. In a recent matched control study of neutropenic hematologic patients, ceftolozane/tazobactam exhibited a significant lower mortality rate for *P. aeruginosa* BSI compared with other therapeutic regimens, including those for DTR strains [83^▪^].

Despite limited data, imipenem/relebactam has shown promising activity against DTR *P. aeruginosa* in the RESTORE-IMI-1 trial, as well as in early real-life experiences [58,59], especially in the case of AmpC hyperproduction [55,57,84].

Ceftazidime/avibactam could be valid treatment option for DTR *P. aeruginosa*, as demonstrated by a pooled study that combined all available clinical trial data, which indicated a favourable clinical response [85]. In particular, when ceftolozane/tazobactam resistance is present because of carbapenemases production, ceftazidime/avibactam should be considered, even combined with aztreonam if MBL is expressed [86].

Cefiderocol is another important option to consider in this setting, both for DTR *P. aeruginosa* and in the case of MBL expressed. It demonstrated noninferiority compared with best available therapy in terms of mortality, clinical cure and microbiological persistence in the CREDIBLE trial considering DTR *P. aeruginosa* infections [60^▪^,^87^]. In real-life experience, it showed good results, with a clinical cure rate of 70% in DTR *P. aeruginosa* infections with previous failed treatment and a survival rate of 58% in different type of infections because of MBL *P. aeruginosa* strains [88,89].

In our opinion, colistin-based regimens should be considered as a last option, especially when new BL/BLI are not available, in case of severe beta-lactam allergies or when new BL/BLI resistance is present [90]. Despite the lack of randomized clinical trials and the limited and biased real-life experience data, combination treatment with new BL/BLI regimens, especially for critically ill patients in the ICU, could be considered as empirical therapy to reduce the risk of treatment failure while waiting for susceptibility results. This approach should be reserved for selected cases, and the possible partner drugs for DTR-GNB could include fosfomycin, polymyxins, or aminoglycosides [43^▪^–45^▪^,^91^].

### Difficult-to-resistance *Acinetobacter baumannii*

*A. baumannii* is a challenging pathogen, because of its strong capacity to resist to multiple antibiotics through different mechanisms such as efflux pumps, modifications in antibiotic binding sites carbapenemase expression. Class D carbapenemases represent the predominant class, including OXA-23-like, OXA-24/40-like, OXA-58- like but also class B and A could be acquired [92–95].

Currently, there is limited data available to recommend a specific therapy for severe infections because of DTR *A. baumannii*. Polymyxin is still considered the backbone against this pathogen with recent meta-analysis showing improved clinical response with this regimen [96]. Ampicillin/sulbactam is generally recommended as part of the treatment regimen if in-vitro activity is confirmed, and combination with two in-vitro active antibiotics should be considered [44^▪^,45^▪^]. Potential antibiotic combinations could include high-dose tigecycline, aminoglycosides or fosfomycin depending on the site of infection. A recent case series, reported successful treatment with a salvage triple combination therapy [97].

Cefiderocol is currently the only new option for DTR *A. baumannii* infections. Even though it showed disappointing results in the registry trial, real-life experience demonstrated promising data, especially in BSI [98–100], making cefiderocol a valid treatment option for DTR *A. baumannii* infections.

Lastly, eravacycline has also shown good in-vitro activity but clinical data is still lacking [101^▪^].

## SOURCE CONTROL

Another important aspect to consider is the control of the source of infection. Along with antimicrobial therapy, source control is critical for achieving clinical success. In community-acquired infections, this typically involves a surgical approach for obstructive UTIs, IAI, or skin and soft tissue infections. In a hospital setting, it often requires the removal of vascular devices and debridement of surgical site infections [18,102]. Failure to achieve source control, especially in severe IAI, has been associated with increased mortality in several studies [10,23,30]. The optimal timing of intervention is unknown, but efficacy is time-dependent, especially in severe IAI [103–105]. Moreover, without an aggressive approach, the inoculum effect and the biofilm production of GNB may lead to a failure in infection control and poor outcomes for the patient.

## CONCLUSION

DTR-GNB infections pose a significant threat to patient outcomes, especially in the ICU setting and are associated with high morbidity and mortality rates. However, recent advances in treatment options have led to the approval of novel therapies with good safety profile and high clinical success, as evidenced by real-life experiences. Despite this, *A. baumannii* infections continue to present limited therapeutic options, highlighting the need for further efforts in this area.

It is important for clinicians to use new antibiotics appropriately, as pathogens resistant to newer options such as ceftolozane/tazobactam, ceftazidime/avibactam, and cefiderocol have already been identified, creating a new challenge in the management of severe infections. Further clinical data from randomized trial and real-life experience are necessary to guide the proper use of these antibiotics.

## Acknowledgements

*None.*

### Financial support and sponsorship

*None.*

### Conflicts of interest

*M.B. has participated in the past 5 years in advisory boards, research grants and/or received speaker bureau from Astellas, Pfizer, MSD, Gilead, Angelini, Bayer, Biomerieux, Cidara, Menarini, and Shionogi. The other authors declare no conflicts of interest.*

## REFERENCES AND RECOMMENDED READING

Papers of particular interest, published within the annual period of review, have been highlighted as:

▪ of special interest
▪▪ of outstanding interest
